# Biochemical Changes in Anterior Chamber of the Eye in Diabetic Patients—A Review

**DOI:** 10.3390/jcm13092581

**Published:** 2024-04-27

**Authors:** Joanna Dolar-Szczasny, Agnieszka Drab, Robert Rejdak

**Affiliations:** 1 Department of General and Pediatric Ophtalmology, Medical University of Lublin, 20-079 Lublin, Poland; oko@usk1.pl; 2 Department of Medical Informatics and Statistics with e-Health Lab, Medical University of Lublin, 20-954 Lublin, Poland; agnieszkabaranska@umlub.pl

**Keywords:** aqueous humor, anterior chamber, diabetic retinopathy, advanced glycation end products, diabetic macular edema

## Abstract

This article aims to provide a comprehensive review of the biochemical changes observed in the anterior chamber of the eye in diabetic patients. The increased levels of inflammatory markers, alterations in antioxidant defense mechanisms, and elevated levels of advanced glycation end products (AGEs) in the aqueous humor (AH) are explored. Additionally, the impact of these biochemical changes on diabetic retinopathy progression, increased intraocular pressure, and cataract formation is discussed. Furthermore, the diagnostic and therapeutic implications of these findings are presented. This study explores potential biomarkers for detecting diabetic eye disease at an early stage and monitoring its progression. An investigation of the targeting of inflammatory and angiogenic pathways as a potential treatment approach and the role of antioxidant agents in managing these biochemical changes is performed.

## 1. Introduction

Diabetes mellitus (DM) is a chronic metabolic disorder characterized by elevated blood glucose levels due to impaired insulin production or utilization. This condition affects millions of individuals worldwide and is associated with various complications, including cardiovascular disease, neuropathy, and retinopathy [1,2]. Diabetic retinopathy (DR), in particular, is a leading cause of blindness among working-age adults [3].

The anterior chamber of the eye, which includes the cornea, iris, and lens, is responsible for regulating intraocular pressure and maintaining the clarity of the visual pathway.

The clear, watery fluid known as the aqueous humor (AH) occupies the space between the cornea and the lens in the front part of the eye. The composition of AH is a topic of great interest to researchers and medical professionals alike. It is primarily composed of water, electrolytes, and proteins. The precise balance of these components is essential for maintaining the optical properties of the eye and ensuring its normal functioning. Any disruption in the composition of this fluid can lead to various eye conditions and diseases.

The main component of the AH—water—accounts for approximately 98% of its total volume. Various disease conditions can have a profound impact on the composition of the AH. For instance, conditions like glaucoma, uveitis, and diabetic retinopathy can alter the balance of electrolytes and proteins within the anterior chamber of the eye.

The AH fulfills several important functions in the eye. First and foremost, it provides nourishment to the avascular tissues of the cornea and the lens. Since the cornea lacks blood vessels, it relies on the AH for the supply of oxygen and nutrients. Furthermore, the AH plays a crucial role in preserving the spherical structure of the eyeball. It exerts a gentle pressure, known as intraocular pressure (IOP), which keeps the eyeball in its spherical form. This pressure is essential for the proper functioning of the eye and relies on a precise equilibrium between the generation and outflow of waste products from the avascular tissues of the eye. It transports and eliminates waste substances, such as lactic acid and carbon dioxide, ensuring a healthy environment for optimal eye function [4].

The AH is continuously generated by the ciliary body, a structure located behind the iris. The ciliary body contains specialized cells called ciliary epitheliums, which actively secrete the aqueous humor into the posterior chamber of the eye. From there, it flows into the anterior chamber through the pupil. The production of AH is a dynamic process regulated by various factors, including hormones, neurotransmitters, and local tissue factors. The rate of production is influenced by the needs of the eye and the metabolic demands of its various tissues [5]. Despite not having straight interaction with the retina, the AH can receive proteins from posterior pole structures through the blood–retinal barrier and cilio-retinal circulation in DR [6]. Additionally, these proteins can also diffuse through the vitreous humor (VH) and the VH–AH barrier [7,8,9]. Consequently, the AH includes proteins that may serve as indicators of the advancement of DR in individuals with DM.

The pathophysiology of DR involves several interconnected mechanisms. Firstly, chronic hyperglycemia damages the endothelium of the blood vessels, leading to the formation of microaneurysms, capillary blockage, and the leakage of fluid and blood into the retina. These events can result in reduced blood flow and inadequate oxygen supply to the retinal tissue, causing ischemia and hypoxia. To counterbalance deficiency, the retina releases a range of growth factors that stimulate the development of new blood vessels (neovascularization).

Complications associated with DR encompass diabetic macular edema (DME), proliferative diabetic retinopathy (PDR), cataracts, retinal detachment, and glaucoma.

Understanding the biochemical alterations in the anterior chamber of the eye in diabetic patients is essential for comprehending the pathophysiology of diabetic eye disease and developing effective diagnostic and therapeutic strategies. Moreover, this knowledge may contribute to the development of more accurate diagnostic tools, allowing for early intervention and improved visual outcomes for individuals with diabetes.

## 2. Biochemical Changes in Anterior Chamber

The anterior chamber of the eye, the space between the cornea and the iris, plays a crucial role in maintaining the overall health and functionality of the eye. In diabetic patients, however, this delicate balance can be disrupted by various biochemical changes that occur as a result of their disease. Ample evidence has been gathered regarding notable alterations in the composition of the AH among individuals with diabetes. The analysis of AH samples is crucial in comprehending the pathophysiology of diabetic eye complications and developing effective diagnostic and therapeutic strategies.

### 2.1. Methods of AH Analysis

For diagnostic purposes, the collection of AH samples is usually performed through a paracentesis procedure in patients who are undergoing different anterior segment surgeries or in post-mortem cases. The quantities of liquid collected are minimal, ranging from 100 to 150 µL [10,11]. Once obtained, AH samples are typically stored undiluted directly at −80 °C. Analyzing these samples can provide valuable information, but the collection process carries inherent risks that should be considered [12]. Conversely, post-mortem eye samples exhibit distinct characteristics resulting from the buildup of metabolic byproducts and unregulated post-death phenomena. Therefore, fluids obtained from living individuals are regarded as more beneficial.

The techniques employed for the analysis of AH include spectroscopic, chromatographic, and mass spectrometry methods [13]. Spectroscopic techniques, such as UV–visible, infrared, and Raman spectroscopy, allow for the characterization of molecular composition and structure. Chromatographic techniques, including HPLC (high-performance liquid chromatography), GC (gas chromatography), and TLC (thin-layer chromatography), enable the separation and identification of different compounds. Mass spectrometry, including ESI-MS (electrospray ionization–mass spectrometry), MALDI-MS (matrix-assisted laser desorption/ionization–mass spectrometry), and GC-MS (gas chromatography–mass spectrometry), offers high sensitivity and selectivity for the identification and quantification of various analytes.

### 2.2. Increased Levels of Inflammatory Markers

Chronic low-grade inflammation, known as sterile inflammation, is now considered a key component of diabetic eye disease. This is due to one of the prominent biochemical changes observed in the anterior chamber of diabetic patients, which is the elevation of inflammatory markers. These markers include cytokines, chemokines, and adhesion molecules, which are molecules responsible for the initiation and regulation of the inflammatory response (Table 1).

#### 2.2.1. Cytokines

Cytokines, which function as signaling molecules within the immune system, are compact proteins, regulating inflammation and immune responses. Numerous cytokines have been detected within the anterior chamber of patients with diabetic retinopathy, each with its own distinct role in the disease process.

One of the most well known is interleukin-6 (IL-6), which is a pro-inflammatory cytokine that has been found to be elevated in the AHs of individuals with diabetic retinopathy. Increased IL-6 levels are linked to an increased risk of disease advancement and severity. The available information indicates that IL-6 can have a role in causing inflammation and vascular leakage, which may then lead to the occurrence of retinal pathologies [14,15,16,17,18].

Tumor necrosis factor-alpha (TNF-α) is another pro-inflammatory cytokine that has been found to be elevated in the AHs of patients with DR. TNF-α promotes inflammation and the impairment of vascular function, leading to the disruption of the blood–retinal barrier. Its presence in the AH suggests its involvement in the pathogenesis of DR [14,15,16,17,18].

Another cytokine—interleukin-1-beta (IL-1β)—plays a significant role as a highly effective cytokine that promotes inflammation, and it has been shown to be upregulated in the AHs of individuals with DR. IL-1β promotes the synthesis of additional agents that provoke inflammation and induces vascular endothelial cell apoptosis, contributing to the development of retinal damage [15,16,17,18].

Various independent research studies regarding alterations in the levels of other cytokines in the AHs of individuals with diabetes have been published. The additional cytokines that have been investigated include IL-10 (interleukin-10), IL-12 (interleukin-12), IP-10 (interferon gamma-induced protein 10), PEDF (pigment epithelium-derived factor), PGF (placental growth factor), TGF-β (transforming growth factor-beta), bFGF (basic fibroblast growth factor), HGF (hepatocyte growth factor), and CCL5 (chemokine ligand 5) [17,19,20,21,22,23]. Nevertheless, some of these findings remain ambiguous. The situation is similar for the determination of biomarkers, including α-1-acid glycoprotein (AGP), apolipoprotein A-I (APOA1), and apolipoprotein A-IV (APOA4) [24,25].

An extensive investigation of the AH composition revealed that there were nearly 200 proteins that display varying levels of expression between individuals with diabetes and those without the condition. These proteins are primarily associated with secretory pathways, making them valuable as biological markers in both clinical studies and fundamental analysis. When comparing subjects with diabetes to control subjects, it was observed that only some protein pathways displayed an increase or decrease in activity. The most prominently represented pathways in PDR subjects were complement and coagulation cascades, the phosphoinositide 3-kinase (PI3K-Akt) signaling pathway, and cholesterol metabolism [26].

#### 2.2.2. Chemokines

Chemokines, which are compact signaling proteins, have a vital function in the immune system’s cell migration and recruitment to sites of inflammation. Several chemokines have been discovered to be increased in the AHs of patients diagnosed with DR, indicating their involvement in the inflammatory processes underlying this disease. The most important are monocyte chemoattractant protein-1 (MCP-1), interleukin-8 (IL-8), and macrophage inflammatory protein-1-alpha (MIP-1α) [17,27].

MCP-1 is a chemokine that attracts monocytes to locations where inflammation occurs. Increased levels of MCP-1 have been detected in the anterior chamber of diabetic patients, suggesting a role in the recruitment of inflammatory cells to the retina and the development of retinal inflammation [28,29].

Another chemokine is IL-8, which promotes the migration and activation of neutrophils. Elevated levels of IL-8 have been found in the AHs of individuals with DR, indicating its involvement in the recruitment and activation of neutrophils in the retina, leading to retinal damage [18,28,29,30].

MIP-1α is a chemokine that attracts and activates macrophages, which are essential immune cells involved in the inflammatory response. Increased levels of MIP-1α have been observed in the anterior chambers of patients with DR, suggesting its contribution to the recruitment and activation of macrophages in the retina, exacerbating retinal inflammation [30,31].

#### 2.2.3. Adhesion Molecules

Adhesion molecules are proteins that facilitate the attachment and migration of immune cells to the endothelial cells lining the blood vessels. Several adhesion molecules have been identified in the AHs of diabetic retinopathy patients, indicating their role in the inflammatory processes associated with this disease. The most supported by evidence are vascular cell adhesion molecule-1 (VCAM-1) and intercellular adhesion molecule-1 (ICAM-1).

VCAM-1 is an adhesion molecule that plays a crucial role in the recruitment of immune cells to sites of inflammation. Increased levels of VCAM-1 have been detected in the aqueous humor of individuals with DR, suggesting its involvement in the adhesion and migration of immune cells to the retinal vasculature, contributing to retinal inflammation [31].

ICAM-1 is another molecule that facilitates the attachment and migration of immune cells to the endothelial cells. Elevated levels of ICAM-1 have been observed in the anterior chambers of diabetic patients, indicating its role in the recruitment and activation of immune cells in the retina, leading to retinal damage [31].

### 2.3. Alterations in Antioxidant Defense Mechanisms of the AH

Diabetes is characterized by increased oxidative stress, which arises from an imbalance between the production of reactive oxygen species (ROS) and the body’s antioxidant defense mechanisms [32]. In the anterior segment of the eyes of diabetic patients, there is a disruption in the normal antioxidant defense system, resulting in the decreased activity of antioxidant enzymes such as superoxide dismutase (SOD), catalase (CAT), and glutathione peroxidase (GPx) [33,34].

SOD is an essential enzymatic antioxidant found in the AH. It acts by catalyzing the conversion of superoxide radicals into less harmful hydrogen peroxide and molecular oxygen. The enzymatic activity of SOD helps prevent the accumulation of superoxide radicals, which exhibit a significant level of reactivity and have the potential to cause extensive harm to ocular tissues [35,36].

GPx is another crucial antioxidant enzyme present in the aqueous humor. It plays a vital role in reducing hydrogen peroxide and organic hydroperoxides to their respective alcohols, thus preventing oxidative damage. GPx is particularly effective in neutralizing lipid hydroperoxides, which can be detrimental to the integrity of cell membranes [37,38].

CAT is an enzyme found in AH that plays a critical role in the breakdown of hydrogen peroxide into water and molecular oxygen. By doing so, catalase helps prevent the accumulation of hydrogen peroxide, which can induce oxidative stress and damage cellular components. This enzymatic antioxidant activity is essential for maintaining the overall redox balance within AH, and compromised antioxidant capacity further exacerbates oxidative stress, leading to cellular damage and contributing to the pathological processes in DR [39,40,41].

In addition to enzymatic antioxidants, the AH also contains various non-enzymatic antioxidants that contribute to its overall antioxidant defense mechanisms. The most well-known ascorbic acid (vitamin C) is a water-soluble antioxidant present in the anterior chamber. It acts as a potent scavenger of free radicals, neutralizing them and preventing oxidative damage to ocular tissues. Ascorbic acid also regenerates vitamin E, another important antioxidant, enhancing its antioxidant capacity within the anterior chamber. Vitamin E encompasses a group of fat-soluble antioxidants, including tocopherols and tocotrienols. These antioxidants protect cell membranes from oxidative damage by scavenging lipid peroxyl radicals. Within the AH, vitamin E works synergistically with other antioxidants to maintain a balanced redox environment. Both vitamins are found in altered concentrations in the anterior chambers of diabetic patients [42,43,44,45].

Another antioxidant—glutathione—is a tripeptide antioxidant present in high concentrations in the AH. It acts as a potent reducing agent, directly neutralizing reactive oxygen species and reactive nitrogen species. Glutathione also plays a crucial role in the regeneration of other antioxidants, such as vitamin C and vitamin E, further enhancing the overall antioxidant defense mechanisms within the anterior chamber [46]. This ingredient has different concentrations in patients with diabetes in plasma, the AH, and the vitreous [47,48].

#### Regulation of Antioxidant Defense Mechanisms

The antioxidant defense mechanisms within the anterior chamber are tightly regulated to ensure their optimal function in combating oxidative stress. A central role in regulating the expression of several antioxidant enzymes and proteins is played by nuclear factor erythroid 2-related factor 2 (Nrf2), which is a transcription factor. The activation of Nrf2 induces the expression of genes that encode antioxidant enzymes, such as SOD, GPx, and CAT, thereby enhancing the antioxidant capacity of the AH in the eye.

The activity of Nrf2 is controlled by a cytoplasmic protein: Kelch-like ECH-associated protein 1 (Keap1). Under normal conditions, Keap1 binds to Nrf2 and promotes its degradation, keeping the antioxidant defense mechanisms in check [49,50]. However, when exposed to oxidative stress, Keap1 is modified, resulting in the release and stimulation of Nrf2, which subsequently triggers the upregulation of antioxidant enzymes [51,52,53,54,55,56].

The disproportion between the generation of reactive oxygen species and the antioxidant protective mechanisms present in diabetes can disrupt the delicate redox balance in AH. This imbalance can activate diverse signaling pathways, such as the nuclear factor kappa B (NF-κB) pathway, which can further influence the expression of antioxidant enzymes and proteins. Understanding the intricate regulation and interplay of enzymatic and non-enzymatic antioxidant defense mechanisms within the AH is crucial for unraveling the pathogenesis of DR and developing targeted therapeutic strategies [57].

### 2.4. Elevated Levels of Advanced Glycation End Products (AGEs)

Advanced glycation end products (AGEs) are a diverse group of compounds formed through non-enzymatic interactions that occur between reducing sugars and proteins or lipids. In the anterior chambers of diabetic patients, there is an accumulation of AGEs due to sustained hyperglycemia. These AGEs can induce inflammation, oxidative stress, and tissue remodeling, all of which contribute to the occurrence and progression of diabetic eye complications. Furthermore, AGEs are involved in the cross-linking of proteins within anterior chamber structures.

The process by which AGEs are formed is known as glycation, and it occurs naturally in the human organism as a part of regular metabolism. However, in conditions such as diabetes, elevated levels of blood glucose can cause the excessive formation of AGEs. The formation of AGEs begins with a reaction between a reducing sugar, such as glucose or fructose, and a free amino group in a protein or another biomolecule. This initial glycation reaction results in the formation of Schiff bases, which are reversible and relatively unstable. Over time, Schiff bases undergo further rearrangements and modifications, leading to the formation of more stable and irreversible AGEs. These AGEs can accumulate in various tissues, including the eye, and are involved in the pathogenesis of diabetic complications [58,59,60]. The build-up of AGEs in tissues can lead to the cross-linking of proteins and the formation of abnormal structures, which can impair tissue function. Furthermore, AGEs have the ability to bind with a specific cell surface receptor known as receptor for AGE (RAGE). The attachment of AGEs to RAGE initiates a series of cellular processes, including the generation of pro-inflammatory molecules and oxidative stress. These processes can contribute to the development of chronic eye inflammation and tissue damage, which are characteristic features of diabetes and its complications, including diabetic retinopathy, cataracts, and glaucoma. In DR, AGEs have been shown to contribute to the disruption of the blood–retinal barrier, which plays a vital role in maintaining the normal function of the retina. AGE-induced inflammation and oxidative stress can also promote the development of retinal vascular abnormalities, leading to retinal ischemia and pathological neovascularization. Additionally, AGEs can impair the function of RPE cells, which play a vital role in the maintenance of retinal integrity. In cataracts, the accumulation of AGEs in the lens proteins can lead to protein cross-linking, resulting in lens opacification and visual impairment. AGE-induced oxidative stress and inflammation have also been suggested to be contributing factors in the development of glaucoma, a progressive optic neuropathy characterized by the degeneration of retinal ganglion cells [61].

#### Angiogenic Factors

The development of abnormal blood vessels, known as neovascularization, is a hallmark of proliferative DR and neovascular glaucoma—the late phase of DR. Pathological vessels can be located in the posterior pole of the eye, either on the optic disc (NVD—new vessels on the optic disc) or elsewhere (NVE—new vessels elsewhere) and also in the anterior chamber of the eye on the iris and the iridocorneal angle. The angiogenic process is regulated by the complex interplay of pro- and anti-angiogenic factors. Several studies have explored the presence of angiogenic factors in the AHs of diabetic patients, including vascular endothelial growth factor (VEGF) 17, angiopoietin-2 (Ang-2), platelet-derived growth factor (PDGF), and pigment epithelium-derived factor (PEDF). These factors have shown potential as biomarkers for monitoring disease progression and evaluating the response to anti-angiogenic therapies [27,62].

VEGF is a primary regulator of the angiogenesis process and is known to play a pivotal role in ocular neovascularization. Increased levels of VEGF have been observed in ocular diseases such as age-related macular degeneration (AMD), DR, and retinopathy of prematurity. Quantifying VEGF levels in the AH can provide valuable information about disease severity and responses to treatment [63]. While VEGF has received significant attention, it is important to acknowledge that other angiogenic factors also contribute to the angiogenic process in diabetic patients. Fibroblast growth factor (FGF) and insulin-like growth factor (IGF) have been shown to promote neovascularization and have an impact on the pathological changes observed in DR. Research has indicated that these factors interact with each other and with VEGF, forming a complex network of signaling pathways that regulate angiogenesis [27].

PDGF is another important angiogenic factor involved in ocular angiogenesis. It has a function in the recruitment and proliferation of pericytes, which are essential for the stability of blood vessels. Abnormal PDGF signaling has been linked to several ocular disorders, including DR. Measuring PDGF levels in the AH has the potential to provide valuable insights into the mechanisms underlying angiogenic processes in the eye. IGF, being a potent mitogen that stimulates the growth and viability of cells, is an additional aspect to consider. In the eye, IGF has been shown to stimulate endothelial cell proliferation and neovascularization. Elevated levels of IGF in the anterior chamber of the eye have been linked to retinal neovascularization in diseases such as proliferative DR [64]. Assessing IGF levels in the AH can shed light on the role of this factor in ocular angiogenesis.

Moreover, there are intricate connections between growth factors and inflammation-related factors. VEGF expression, for instance, is stimulated by IL-6 [65], while the expression of other cytokines and chemokines is increased by IL-8 [66]. Previous research conducted on individuals with DME has found a notable association between levels of VEGF in the vitreous and IL-6 [67]. Additionally, one study discovered noteworthy complex associations among IL-6, IL-8, IL-18, MCP-1, and TNF-α in the AHs of DME patients; however, these correlations were not entirely consistent [15].

Further research has established that VEGF levels exhibit a negative correlation with IL-12 and TNF-α in the AH. In one study, in a diabetic group, there was a decrease in the levels of IL-2, IL-10, IL-12, and TNF-α in contrast to a control group consisting of individuals without diabetes, which may be attributable to increased VEGF levels in the diabetic group. IL-10, IL-12, and TNF-α are significant mediators of innate immunity and also play a role in the adaptive immune response. A decrease in IL-12 is probably responsible for reduced IL-2 in patients with diabetes. It is hypothesized that the presence of low levels of these cytokines in eyes without diabetes offers safeguards against possible damage. Disturbance in this immune balance is probably responsible for certain pathological alterations observed in diabetic eyes [18].

AH analysis has also revealed decreased levels of interferon-alpha (IFN-α) and granulocyte–macrophage colony-stimulating factor (GM-CSF) in diabetic patients in comparison with controls. IFN-α is a strong inhibitor of angiogenesis and has demonstrated effectiveness in the treatment of vascular neoplasms. Two preliminary studies have indicated that IFN-α might be involved in the regression of PDR and provide protection against retinal hemorrhage [68,69]. The present findings, which indicate lower levels of IFN-α in individuals with diabetes, provide additional evidence to suggest that this cytokine might play a beneficial role in preventing retinal damage associated with diabetes. GM-CSF is associated with retinal changes in uveitis [70]. This cytokine plays a crucial role in regulating the behavior and function of macrophages, granulocytes, and dendritic cells. TNF-α and interferon-gamma (IFN-γ) have been proven to influence the production of GM-CSF in retinal pigment epithelial cells. Nevertheless, there is a lack of comprehensive knowledge regarding the contribution of GM-CSF to the progression of DR [71].

## 3. Impact of Biochemical Changes

The biochemical alterations that occur in the anterior chamber of the eye in diabetic patients contribute to the development and progression of DR, increased IOP, and cataract formation.

### 3.1. Correlation with Diabetic Retinopathy Progression

DR is a serious complication of diabetes that affects the blood vessels in the retina, leading to vision loss if left untreated. The biochemical changes in the anterior chamber play a key role in the development and progression of this sight-threatening condition. Increased levels of inflammatory markers, such as cytokines and chemokines, contribute to the disruption of the blood–retinal barrier and the recruitment of immune cells into the retina. These processes promote the development of pathological blood vessels and the leakage of fluid leading to DME formation, ultimately causing disorganization in retinal layers and vision impairment [66,72]. The end stage of proliferative DR is tractional retinal detachment.

### 3.2. Association with Increased Intraocular Pressure

Elevated intraocular pressure is a frequent finding in diabetic patients and is associated with an increased risk of developing glaucoma, a leading cause of irreversible blindness [73]. The biochemical changes in the anterior chamber, particularly alterations in antioxidant defense mechanisms, contribute to the imbalance between the production and clearance of reactive oxygen species. This oxidative stress can damage the trabecular meshwork, the tissue responsible for regulating the outflow of AH, leading to impaired drainage and increased intraocular pressure.

Excessive glycation, a non-enzymatic reaction that occurs in diabetic patients, is responsible for the production of several AGEs such as pentosidine, Nε-(carboxymethyl)lysine (CML), and Nε-(carboxyethyl)lysine (CEL). These AGEs tend to accumulate in different tissues, including the eye [61,74,75]. In the context of AH, AGE accumulation can occur through several mechanisms. Firstly, AH is in direct contact with various structures of the eye, such as the lens, cornea, and iris. These structures are exposed to high levels of glucose, which can lead to increased glycation reactions and subsequent AGE formation. AH is continuously replenished through the ciliary body, a structure responsible for the production and secretion of this fluid. Studies have shown that the ciliary body itself can produce AGEs, contributing to their accumulation in AH. Furthermore, the ciliary body expresses receptors for AGEs, suggesting a potential feedback loop where AGEs can further stimulate their own accumulation [58,59,74].

The presence of AGEs in AH has been found to have significant effects on its dynamics [61]. AGEs can increase the viscosity of the aqueous humor, impairing its flow and drainage. This alteration in fluid dynamics can lead to increased intraocular pressure, a known risk factor for the development of glaucoma [73]. Moreover, AGEs have been shown to provoke oxidative stress and inflammation in the eye. These processes can further contribute to the impairment of dynamics in the anterior chamber and damage to ocular tissues.

### 3.3. Cataract Formation

Inflammatory mediators released in response to AGE accumulation can promote the formation of cataracts, among other age-related ocular conditions.

Cataracts are characterized by the clouding of the lens, which affects vision and can ultimately result in loss of vision if left untreated. AGEs have been found to accumulate in the lens with age, and their levels are considerably elevated in lenses affected by cataracts in comparison with clear lenses [33,34,74,76,77]. The presence of AGEs in the lens can disrupt its structure and, consequently, function. AGEs can cross-link proteins within the lens, resulting in the development of protein aggregates and the loss of lens transparency. Additionally, AGEs have the potential to trigger oxidative stress and inflammation in lens cells, causing further damage to lens proteins and impairing their ability to maintain lens clarity [74,76,77]. All research data support the observation that cataracts are more common and tend to develop at an earlier age in diabetic patients as opposed to the overall population.

Overall, the biochemical changes occurring in the anterior chamber of the eye in diabetic patients have profound implications for their ocular health. By unraveling the impact of these changes, we can identify potential biomarkers for diabetic eye diseases, develop strategies to target inflammatory pathways for treatment, and explore the role of antioxidant agents in disease management. Through continued research and clinical advancements, we can strive to improve the quality of life of individuals living with diabetes and reduce the burden of diabetic eye complications.

## 4. Clinical Implications

There is no evidence to suggest that any particular anti-diabetic treatment can have a positive impact on the onset or progression of diabetic retinopathy (DR) without addressing the improvement of blood glucose levels. However, understanding the presence and severity of DR in a diabetic patient has implications for treatment. In fact, there is growing evidence indicating that the rapid control of blood glucose levels can actually worsen DR. While this effect is well recognized in advanced-stage DR, it is not as apparent in early-stage DR [78].

### 4.1. Targeting Inflammatory Pathways for Treatment

Biochemical changes in the anterior chamber, particularly increased levels of inflammatory markers, highlight the importance of targeting inflammatory pathways for the treatment of diabetic eye diseases. Inflammation has a significant impact on the development and progression of ocular complications in diabetic patients. Therefore, interventions aimed at reducing inflammation could potentially decelerate or even nearly stop the progression of DR and other ocular complications.

Current therapeutic strategies targeting inflammatory pathways involve the administration of nonsteroidal anti-inflammatory drugs and corticosteroids. The objective of these interventions is to mitigate the inflammatory reaction in individuals with diabetes and hinder the occurrence of eye-related complications.

The use of anti-inflammatory drugs, both systemic and local, has also been explored as a means to target inflammatory pathways in DR. However, more research is needed to determine specific targets within the inflammatory cascade that can be effectively modulated to achieve optimal therapeutic outcomes. These drugs aim to reduce the overall inflammatory burden primarily in the retina and alleviate the associated damage. Nonsteroidal anti-inflammatory drugs (NSAIDs), corticosteroids, and immunosuppressive agents are among the classes of drugs that have been investigated. While many of these drugs may have potential benefits in reducing inflammation, their use is often limited by systemic or local side effects and the need for long-term administration. To surpass these limitations, researchers are currently exploring the advancement of targeted drug delivery systems, such as intravitreal injections or implantable devices, to deliver anti-inflammatory drugs directly to the eye. This approach allows for localized delivery, minimizing systemic exposure and potential side effects.

#### 4.1.1. Corticosteroids

Corticosteroids are the prevailing choice in ophthalmology when it comes to anti-inflammatory treatments. By administering corticosteroids directly to the eye (intravitreal use), it is possible to achieve high concentrations of the drug in the targeted area. However, it is important to note that intravitreal corticosteroid injections come with a high risk of ocular complications, such as glaucoma and cataract formation [79]. Consequently, treatment with intravitreal corticosteroids is typically limited to patients with persistent or refractory diabetic macular edema (DME), especially those with pseudophakic eyes [80]. In addition to their ability to counteract the effects of proinflammatory cytokines, corticosteroids have also been found to provide neuroprotection, as demonstrated in both experimental [81] and clinical studies [82]. Furthermore, they possess antiangiogenic properties [83]. The various impacts of corticosteroids have generated greater attention toward these conventional drugs, resulting in the creation of sustained-release formulations and implants that decrease the need for intravitreal injections [79].

#### 4.1.2. Role of Antioxidant Agents in Disease Management

Oxidative stress is one of the key points in the development of DR. In order to address this issue, researchers have been investigating the potential therapeutic implications of various antioxidant phytochemicals and drugs. These substances have shown promise in controlling and alleviating oxidative stress in the pathogenesis of DR.

Polyphenols, such as epigallocatechin-3-gallate (EGCG), present in green tea, have been found to have strong antioxidant properties. EGCG not only eliminates retinal reactive oxygen species (ROS) but also provides neuroprotection and improves hyperglycemia-induced impairments. It can also suppress inflammatory factors and prevent the formation of advanced glycation end products (AGEs) [84,85,86].

Quercetin, a plant-derived flavonoid, has shown potential in inhibiting oxidative stress injuries caused by diabetes. It facilitates the expression of important antioxidant enzymes in the retina and inhibits the expression of factors associated with apoptosis and neurodegeneration [87].

Resveratrol, a type of polyphenolic compound that does not belong to the flavonoid group, has shown an ability to provide protection against ocular diseases that are associated with aging. It activates pathways that eliminate intracellular ROS caused by elevated glucose levels and inhibits ROS-induced cell death in retinal cells. Resveratrol additionally inhibits inflammation induced by hyperglycemia and restores insulin levels, attenuating inflammation and damage to the retinal layers during DR [88,89,90,91,92].

Curcumin, a bioactive compound found in Curcuma Longa, has shown restorative potential in preventing DR. It exhibits hypoglycemic, antioxidant, and anti-inflammatory properties, impeding structural degeneration and normalizing abnormal DNA methyltransferase function caused by prolonged exposure to high glucose levels [93,94,95].

In addition to polyphenols, other antioxidants have also shown promise in managing oxidative stress in DR. Lutein, a carotenoid found in leafy vegetables, protects the central part of the retina and photoreceptors from oxidative damage. Astaxanthin, a xanthophyll carotenoid, reduces inflammation, ROS, and apoptosis, while zeaxanthin alleviates retinal oxidative stress and inflammatory responses [96,97,98,99,100,101].

Lipoic acid (LA), an antioxidant derived from sulfur-containing compounds, promotes the expression of genes involved in antioxidant and detoxification processes and demonstrates suppressive properties on nuclear factor-κB function [102,103,104]. Vitamins C and E, as well as other antioxidants, normalize retinal oxidative stress and ameliorate microvascular abnormalities in the initial stages of DR [105]. Trace elements like zinc, copper, manganese, and selenium also play a role in protecting the retina from oxidative damage [106,107,108,109].

#### 4.1.3. Anti-Angiogenic Treatment

Treatment with anti-VEGF agents administered intravitreally is currently the preferred initial approach for clinically significant DME, a severe ocular complication [79]. While both proinflammatory cytokines and VEGF contribute to the development of DME, blocking VEGF is the primary therapeutic strategy rather than targeting inflammation [110]. Clinical trials have provided strong evidence that intravitreal anti-VEGF therapy is more effective than laser therapy in preserving and enhancing vision in patients with DME [63]. Nevertheless, approximately half of DME patients respond insufficiently to anti-VEGF treatment [111]. These results suggest that alternative mechanistic pathways may be involved individually or in combination with VEGF in the development of DME. In a seminal study by Aiello et al., it was found that 36% of patients with proliferative diabetic retinopathy (PDR) did not exhibit elevated VEGF levels in the vitreous body, which explains the unsatisfactory response to anti-VEGF therapy in these individuals [112]. Non-responders to anti-VEGF treatment may experience a more relevant pathogenic role from proinflammatory cytokines/chemokines and other angiogenic agents unconnected to VEGF, such as PDGF, bFGF, HGF, and angiopoietin-2.

Aflibercept is one of the most popular intravitreal anti-VEGF drugs due to its ability to provide a more comprehensive inhibition of angiogenesis compared with anti-VEGF agents alone, like bevacizumab and ranibizumab. Consequently, it may yield superior outcomes for specific patients [110]. Aflibercept acts as a soluble receptor decoy that binds with higher affinity to VEGF-A, VEGF-B, and placental growth factor (PIGF) compared with the body’s inherent receptors. By binding to aflibercept instead of the original receptors, VEGF activity is reduced [113].

The angiopoietin (Ang)/Tie2 signaling pathway, known for its critical role in angiogenesis regulation, was recently identified as a promising therapeutic approach. Numerous clinical trials have proven the effectiveness of pharmacologic and biological mediators targeting the Ang/Tie2 pathway [62]. While the VEGF pathway primarily induces endothelial cell growth and initial network generation, the Ang/Tie2 pathway regulates blood vessel remodeling and maturation during subsequent stages of angiogenesis [114]. In treatment- naïve patients with DME, the combined suppression of angiopoietin-2 and VEGF-A using a new anti-VEGF drug—faricimab—has demonstrated superior efficacy compared with treatment with anti-VEGF agents alone [115].

### 4.2. Laser Therapy

Standard laser therapy has been shown to be effective in DR treatment, although not as much as new intravitreal drugs. There are no data available on the impact of focal laser and panphotocoagulation techniques on biomarkers in the AH. Subthreshold micropulse laser (SMPL) is an innovative method that effectively treats DME while minimizing damage to the surrounding tissue. This technique has been proven to decrease the concentration of VEGF. When evaluating DME treatment-naïve eyes, it was found that the levels of RPE biomarkers varied significantly [116]. SMPL’s mechanism of action may involve deactivating microglial cells and reducing the local inflammatory response associated with diabetes [117,118].

## 5. Conclusions

The biochemical changes that occur in the anterior chamber of the eye in diabetic patients have significant implications for the development and progression of diabetic eye diseases. The elevated levels of inflammatory markers observed in the anterior chamber promote the chronic low-grade inflammation characteristic of diabetic eye disease. This inflammation is a pivotal factor in the pathogenesis of diabetic retinopathy, resulting in damage to retinal blood vessels and subsequent vision loss.

Furthermore, alterations in antioxidant defense mechanisms in the anterior chamber of the eye have been observed in diabetic patients. The disproportion between oxidants and antioxidants is responsible for oxidative stress, which further exacerbates the damage caused by inflammation. The accumulation of advanced glycation end products (AGEs) in the anterior chamber is another important biochemical change that contributes to the development of diabetic eye diseases, including diabetic retinopathy, cataract formation, and increased intraocular pressure.

The impact of these biochemical changes on diabetic eye diseases is substantial. The correlation between the biochemical changes in the anterior chamber and the progression of diabetic retinopathy highlights the importance of early detection and intervention. Additionally, the association between these changes and increased intraocular pressure emphasizes the need for the regular monitoring and management of this risk factor. Moreover, the link between biochemical changes and cataract formation underscores the importance of comprehensive eye care in diabetic patients.

The diagnostic and therapeutic implications of these findings are significant. The identification of potential biomarkers for diabetic eye disease can aid in the early detection and monitoring of disease progression. Targeting inflammatory pathways, such as through the use of anti-inflammatory agents, holds promise for the treatment of diabetic eye diseases. Additionally, the role of antioxidant agents in disease management provides potential avenues for intervention and prevention.

## Figures and Tables

**Table 1:** Key biomarkers responsible for initiating and controlling the inflammatory response in the anterior chamber of the eye in diabetes.

Cytokines	Interleukin-6 (IL-6) Tumor necrosis factor-alpha (TNF-α) Interleukin-1-beta (IL-1β) Interleukin-10 (IL-10) Interleukin-12 (IL-12) Interferon gamma-induced protein 10 (IP-10) Pigment epithelium-derived factor (PEDF) Placental growth factor (PGF) Transforming growth factor-beta (TGF-β) Basic fibroblast growth factor (bFGF) Hepatocyte growth factor (HGF) Chemokine ligand 5 (CCL5)
Chemokines	Monocyte chemoattractant protein-1 (MCP-1) Interleukin-8 (IL-8) Macrophage inflammatory protein-1-alpha (MIP-1α)
Adhesion molecules	Vascular cell adhesion molecule-1 (VCAM-1) Intercellular adhesion molecule-1 (ICAM-1)
Other biomarkers	Alpha-1-acid glycoprotein (AGP) Apolipoprotein A-I (APOA1) Apolipoprotein A-IV (APOA4)

## Data Availability

Not applicable.

## References

[1] Ginter E., Simko V. (2012). Global Prevalence and Future of Diabetes Mellitus. Adv Exp. Med. Biol..

[2] Yau J.W.Y., Rogers S.L., Kawasaki R., Lamoureux E.L., Kowalski J.W., Bek T., Chen S.J., Dekker J.M., Fletcher A., Grauslund J. (2012). Global Prevalence and Major Risk Factors of Diabetic Retinopathy. Diabetes Care..

[3] Trento M., Passera P., Trevisan M., Schellino F., Sitia E., Albani S., Montanaro M., Bandello F., Scoccianti L., Charrier L. (2013). Quality of life, impaired vision and social role in people with diabetes: A multicenter observational study. Acta Diabetol..

[4] Sunderland D.K., Sapra A. (2024). Physiology, Aqueous Humor Circulation. StatPearls [Internet].

[5] Fautsch M.P., Johnson D.H. (2006). Aqueous Humor Outflow: What Do We Know? Where Will It Lead Us?. Investig. Opthalmology Vis. Sci..

[6] DiMattio J. (1991). In vivo use of neutral radiolabelled molecular probes to evaluate blood-ocular barrier integrity in normal and streptozotocin-diabetic rats. Diabetologia.

[7] Klaassen I., Van Noorden C.J.F., Schlingemann R.O. (2013). Molecular basis of the inner blood-retinal barrier and its breakdown in diabetic macular edema and other pathological conditions. Prog. Retin Eye Res..

[8] Thornit D.N., Vinten C.M., Sander B., Lund-Andersen H., la Cour M. (2010). Blood-retinal barrier glycerol permeability in diabetic macular edema and healthy eyes: Estimations from macular volume changes after peroral glycerol. Investig. Ophthalmol Vis. Sci..

[9] Funatsu H., Yamashita H., Noma H., Mimura T., Nakamura S., Sakata K., Hori S. (2005). Aqueous humor levels of cytokines are related to vitreous levels and progression of diabetic retinopathy in diabetic patients. Graefes Arch Clin. Exp. Ophthalmol..

[10] Murthy K.R., Rajagopalan P., Pinto S.M., Advani J., Murthy P.R., Goel R., Subbannayya Y., Balakrishnan L., Dash M., Anil A.K. (2015). Proteomics of Human Aqueous Humor. OMICS.

[11] Goel M. (2010). Aqueous Humor Dynamics: A Review. Open Ophthalmol. J..

[12] Tamhane M., Cabrera-Ghayouri S., Abelian G., Viswanath V. (2019). Review of Biomarkers in Ocular Matrices: Challenges and Opportunities. Pharm. Res..

[13] Pietrowska K., Dmuchowska D.A., Krasnicki P., Mariak Z., Kretowski A., Ciborowski M. (2018). Analysis of pharmaceuticals and small molecules in aqueous humor. J. Pharm. Biomed. Anal..

[14] Gustavsson C., Agardh C., Agardh E. (2013). Profile of intraocular tumour necrosis factor-α and interleukin-6 in diabetic subjects with different degrees of diabetic retinopathy. Acta Ophthalmol..

[15] Hu K.K., Tian C.W., Li M.H., Wu T., Gong M., Wei X.L., Du Y.R., Hui Y.N., Du H.J. (2023). Differential analysis of aqueous humor cytokine levels in patients with macular edema secondary to diabetic retinopathy or retinal vein occlusion. Int. J. Ophthalmol..

[16] Mason R.H., Minaker S.A., Lahaie Luna G., Bapat P., Farahvash A., Garg A., Bhambra N., Muni R.H. (2022). Changes in aqueous and vitreous inflammatory cytokine levels in proliferative diabetic retinopathy: A systematic review and meta-analysis. Eye.

[17] Obadă O., Pantalon A.D., Rusu-Zota G., Hăisan A., Lupuşoru S.I., Constantinescu D., Chiseliţă D. (2022). Aqueous Humor Cytokines in Non-Proliferative Diabetic Retinopathy. Medicina.

[18] Feng S., Yu H., Yu Y., Geng Y., Li D., Yang C., Lv Q., Lu L., Liu T., Li G. (2018). Levels of Inflammatory Cytokines IL-1 β, IL-6, IL-8, IL-17A, and TNF- α in Aqueous Humour of Patients with Diabetic Retinopathy. J. Diabetes Res..

[19] Saucedo L., Pfister I.B., Zandi S., Gerhardt C., Garweg J.G. (2021). Ocular TGF-β, Matrix Metalloproteinases, and TIMP-1 Increase with the Development and Progression of Diabetic Retinopathy in Type 2 Diabetes Mellitus. Mediat. Inflamm..

[20] Khuu L., Tayyari F., Sivak J.M., Flanagan J.G., Singer S., Brent M.H., Huang D., Tan O., Hudson C. (2017). Aqueous humour concentrations of TGF-β, PLGF and FGF-1 and total retinal blood flow in patients with early non-proliferative diabetic retinopathy. Acta Ophthalmol..

[21] Shinoda K., Ishida S., Kawashima S., Wakabayashi T., Matsuzaki T., Takayama M., Shinmura K., Yamada M. (1999). Comparison of the levels of hepatocyte growth factor and vascular endothelial growth factor in aqueous fluid and serum with grades of retinopathy in patients with diabetes mellitus. Br. J. Ophthalmol..

[22] Gverović Antunica A., Karaman K., Znaor L., Sapunar A., Buško V., Puzović V. (2012). IL-12 concentrations in the aqueous humor and serum of diabetic retinopathy patients. Graefe’s Arch. Clin. Exp. Ophthalmol..

[23] Min S.H., Lee T.I., Chung Y.S., Kim H.K. (2006). Transforming Growth Factor-β Levels in Human Aqueous Humor of Glaucomatous, Diabetic and Uveitic Eyes. Korean J. Ophthalmol..

[24] Balaiya S., Zhou Z., Chalam K.V. (2017). Characterization of Vitreous and Aqueous Proteome in Humans with Proliferative Diabetic Retinopathy and Its Clinical Correlation. Proteom. Insights.

[25] Saucedo L., Pfister I.B., Schild C., Zandi S., Garweg J.G. (2022). Aqueous Humor Apolipoprotein Concentration and Severity of Diabetic Retinopathy in Type 2 Diabetes. Mediat. Inflamm..

[26] Xiao H., Xin W., Sun L.M., Li S.S., Zhang T., Ding X.Y. (2021). Comprehensive proteomic profiling of aqueous humor proteins in proliferative diabetic retinopathy. Transl. Vis. Sci. Technol..

[27] Kaštelan S., Orešković I., Bišćan F., Kaštelan H., Gverović Antunica A. (2020). Inflammatory and angiogenic biomarkers in diabetic retinopathy. Biochem. Med..

[28] Chen H., Zhang X., Liao N., Wen F. (2017). Assessment of biomarkers using multiplex assays in aqueous humor of patients with diabetic retinopathy. BMC Ophthalmol..

[29] Dong N., Xu B., Wang B., Chu L. (2013). Study of 27 aqueous humor cytokines in patients with type 2 diabetes with or without retinopathy. Mol. Vis..

[30] Yamakawa N., Komatsu H., Usui Y., Tsubota K., Wakabayashi Y., Goto H. (2023). Immune Mediators Profiles in the Aqueous Humor of Patients with Simple Diabetic Retinopathy. J. Clin. Med..

[31] Song S., Yu X., Zhang P., Dai H. (2020). Increased levels of cytokines in the aqueous humor correlate with the severity of diabetic retinopathy. J. Diabetes Complications..

[32] Behl T., Kaur I., Kotwani A. (2016). Implication of oxidative stress in progression of diabetic retinopathy. Surv. Ophthalmol..

[33] Zoric L. (2003). Some parameters of the oxidative stress in lens, humour aqueous and serum of patients with diabetes and age-related cataract. Srp. Arh. Celok. Lek..

[34] Govindaswamy S., Prabhakar S. (2022). Evaluation of antioxidative enzymes levels and lipid peroxidation products levels in diabetic and non diabetic senile cataract patients. J. Diabetes. Metab. Disord..

[35] Johnson F., Giulivi C. (2005). Superoxide dismutases and their impact upon human health. Mol. Aspects Med..

[36] Kernell A., Lundh B.L., Marklund S.L., Skoog K.O., Björkstén B. (1992). Superoxide dismutase in the anterior chamber and the vitreous of diabetic patients. Investig. Ophthalmol. Vis. Sci..

[37] Pirie A. (1965). Glutathione Peroxidase in Lens and A Source of Hydrogen Peroxide in Aqueous Humour. Biochem. J..

[38] Chalmers R.L. (1989). Hydrogen peroxide in anterior segment physiology: A literature review. Optom. Vis Sci..

[39] Green K., Costarides A.P., Riley M.V. (1990). Role of glutathione in the regulation of anterior chamber hydrogen peroxide. Lens Eye Toxic. Res..

[40] Costarides A.P., Riley M.V., Green K. (1991). Roles of Catalase and the Glutathione Redox Cycle in the Regulation of Anterior-Chamber Hydrogen Peroxide. Ophthalmic. Res..

[41] Csukas S., Costarides A., Riley M.V., Green K. (1987). Hydrogen peroxide in the rabbit anterior chamber: Effects on glutathione, and catalase effects on peroxide kinetics. Curr. Eye Res..

[42] Hsueh Y.J., Chen Y.N., Chen H.C. (2023). Analysis of Total Antioxidant Capacity and Ascorbic Acid in the Aqueous Humor of Patients with Diabetic Retinopathy. Investig. Ophthalmol. Vis Sci..

[43] Park S.W., Ghim W., Oh S., Kim Y., Park U.C., Kang J., Yu H.G. (2019). Association of vitreous vitamin C depletion with diabetic macular ischemia in proliferative diabetic retinopathy. PLoS ONE.

[44] Costagliola C., Iuliano G., Menzione M., Rinaldi E., Vito P., Auricchio G. (1986). Effect of vitamin E on glutathione content in red blood cells, aqueous humor and lens of humans and other species. Exp Eye Res..

[45] Trevithick J.R., Linklater H.A., Mitton K.P., Dzialoszynski T., Sanford S.E. (1989). Modeling cortical cataractogenesis: IX. Activity of vitamin E and esters in preventing cataracts and gamma-crystallin leakage from lenses in diabetic rats. Ann. N. Y. Acad. Sci..

[46] Gumieniczek A., Owczarek B., Pawlikowska B. (2012). Oxidative/Nitrosative Stress and Protein Damages in Aqueous Humor of Hyperglycemic Rabbits: Effects of Two Oral Antidiabetics, Pioglitazone and Repaglinide. Exp. Diabetes Res..

[47] Aldrich B.T., Skeie J.M., Schmidt G.A. (2017). Proteomic Analysis of Aqueous Humor Reveals Progression of Diabetes Mellitus in the Anterior Chamber. Investig. Ophthalmol. Vis Sci..

[48] Kunikata H., Ida T., Sato K., Aizawa N., Sawa T., Tawarayama H., Murayama N., Fujii S., Akaike T., Nakazawa T. (2017). Metabolomic profiling of reactive persulfides and polysulfides in the aqueous and vitreous humors. Sci. Rep..

[49] Bellezza I., Giambanco I., Minelli A., Donato R. (2018). Nrf2-Keap1 signaling in oxidative and reductive stress. Biochim. Biophys. Acta Mol. Cell Res..

[50] Baird L., Yamamoto M. (2020). The Molecular Mechanisms Regulating the KEAP1-NRF2 Pathway. Mol Cell Biol..

[51] Kowluru R.A., Mishra M. (2017). Epigenetic regulation of redox signaling in diabetic retinopathy: Role of Nrf2. Free Radic Biol. Med..

[52] Li X., Deng A., Liu J., Hou W. (2018). The role of Keap1-Nrf2-ARE signal pathway in diabetic retinopathy oxidative stress and related mechanisms. Int. J. Clin. Exp. Pathol..

[53] Zhong Q., Mishra M., Kowluru R.A. (2013). Transcription factor Nrf2-mediated antioxidant defense system in the development of diabetic retinopathy. Investig. Ophthalmol Vis. Sci..

[54] Batliwala S., Xavier C., Liu Y., Wu H., Pang I.H. (2017). Involvement of Nrf2 in Ocular Diseases. Oxid. Med. Cell Longev..

[55] Mishra M., Zhong Q., Kowluru R.A. (2014). Epigenetic modifications of Keap1 regulate its interaction with the protective factor Nrf2 in the development of diabetic retinopathy. Investig. Ophthalmol Vis Sci..

[56] Chen J., Wang Q., Li R., Li Z., Jiang Q., Yan F., Ye J. (2024). The role of Keap1-Nrf2 signaling pathway during the progress and therapy of diabetic retinopathy. Life Sci..

[57] Ohia S.E., Opere C.A., LeDay A.M. (2005). Pharmacological consequences of oxidative stress in ocular tissues. Mutat. Res./Fundam. Mol. Mech. Mutagen..

[58] Kandarakis S.A., Piperi C., Topouzis F., Papavassiliou A.G. (2014). Emerging role of advanced glycation-end products (AGEs) in the pathobiology of eye diseases. Prog. Retin Eye Res..

[59] Bejarano E., Taylor A. (2019). Too sweet: Problems of protein glycation in the eye. Exp. Eye Res..

[60] Yamagishi S.I., Ueda S., Matsui T., Nakamura K., Okuda S. (2008). Role of advanced glycation end products (AGEs) and oxidative stress in diabetic retinopathy. Curr. Pharm. Des..

[61] Shirakami T., Yamanaka M., Fujihara J., Matsuoka Y., Gohto Y., Obana A., Tanito M. (2020). Advanced Glycation End Product Accumulation in Subjects with Open-Angle Glaucoma with and without Exfoliation. Antioxidants.

[62] Whitehead M., Osborne A., Widdowson P.S., Yu-Wai-Man P., Martin K.R. (2019). Angiopoietins in Diabetic Retinopathy: Current Understanding and Therapeutic Potential. J. Diabetes Res..

[63] Simó R., Sundstrom J.M., Antonetti D.A. (2014). Ocular Anti-VEGF Therapy for Diabetic Retinopathy: The Role of VEGF in the Pathogenesis of Diabetic Retinopathy. Diabetes Care..

[64] Sun C., Zhang H., Jiang J., Li Y., Nie C., Gu J., Luo L., Wang Z. (2020). Angiogenic and inflammatory biomarker levels in aqueous humor and vitreous of neovascular glaucoma and proliferative diabetic retinopathy. Int. Ophthalmol..

[65] Didion S. (2017). Cellular and Oxidative Mechanisms Associated with Interleukin-6 Signaling in the Vasculature. Int. J. Mol. Sci..

[66] Noma H., Yasuda K., Shimura M. (2021). Involvement of Cytokines in the Pathogenesis of Diabetic Macular Edema. Int. J. Mol. Sci..

[67] Urbančič M., Petrovič D., Živin A.M., Korošec P., Fležar M., Petrovič M.G. (2020). Correlations between vitreous cytokine levels and inflammatory cells in fibrovascular membranes of patients with proliferative diabetic retinopathy. Mol. Vis..

[68] Leibovitch I., Loewenstein A., Alster Y., Rosenblatt I., Lazar M., Yassur Y., Rubinstein A. (2004). Interferon alpha-2a for proliferative diabetic retinopathy after complete laser panretinal photocoagulation treatment. Ophthalmic. Surg. Lasers Imaging.

[69] Skowsky W.R., Siddiqui T., Hodgetts D., Lambrou F.H., Stewart M.W., Foster M.T. (1996). A pilot study of chronic recombinant interferon-alfa 2a for diabetic proliferative retinopathy: Metabolic effects and opthalmologic effects. J. Diabetes Complicat..

[70] Crane I.J., Kuppner M.C., McKillop-Smith S., Wallace C.A., Forrester J.V. (1999). Cytokine regulation of granulocyte-macrophage colony-stimulating factor (GM-CSF) production by human retinal pigment epithelial cells. Clin. Exp. Immunol..

[71] Cheung C.M.G., Vania M., Ang M., Chee S.P., Li J. (2012). Comparison of aqueous humor cytokine and chemokine levels in diabetic patients with and without retinopathy. Mol. Vis..

[72] Hillier R.J., Ojaimi E., Wong D.T., Mak M.Y., Berger A.R., Kohly R.P., Kertes P.J., Forooghian F., Boyd S.R., Eng K. (2017). Aqueous Humor Cytokine Levels as Biomarkers of Disease Severity in Diabetic Macular Edema. Retina..

[73] Song B.J., Aiello L.P., Pasquale L.R. (2016). Presence and Risk Factors for Glaucoma in Patients with Diabetes. Curr. Diab. Rep..

[74] Franke S., Stein F., Dawczynski J., Blum M., Kubetschka U., Stein G., Strobel J. (2003). Advanced glycation end-products in anterior chamber aqueous of cataractous patients. J. Cataract. Refract. Surg..

[75] Ghanem A.A., Elewa A., Arafa L.F. (2011). Pentosidine and N-carboxymethyl-lysine: Biomarkers for type 2 diabetic retinopathy. Eur. J. Ophthalmol..

[76] Guo Z., Ma X., Zhang R.X., Yan H. (2023). Oxidative stress, epigenetic regulation and pathological processes of lens epithelial cells underlying diabetic cataract. Adv. Ophthalmol. Pract. Res..

[77] Mrugacz M., Pony-Uram M., Bryl A., Zorena K. (2023). Current Approach to the Pathogenesis of Diabetic Cataracts. Int. J. Mol. Sci..

[78] Simó R., Hernández C. (2022). New Insights into Treating Early and Advanced Stage Diabetic Retinopathy. Int. J. Mol. Sci..

[79] Ehlers J.P., Yeh S., Maguire M.G., Smith J.R., Mruthyunjaya P., Jain N., Kim L.A., Weng C.Y., Flaxel C.J., Schoenberger S.D. (2022). Intravitreal Pharmacotherapies for Diabetic Macular Edema. Ophthalmology.

[80] Bandello F., Preziosa C., Querques G., Lattanzio R. (2014). Update of Intravitreal Steroids for the Treatment of Diabetic Macular Edema. Ophthalmic Res..

[81] Zhang X., Lai D., Bao S., Hambly B.D., Gillies M.C. (2013). Triamcinolone Acetonide Inhibits p38MAPK Activation and Neuronal Apoptosis in Early Diabetic Retinopathy. Curr. Mol. Med..

[82] Lynch S.K., Lee K., Chen Z., Folk J.C., Schmidt-Erfurth U., Gerendas B.S., Wahle A., Wykoff C.C., Abràmoff M.D. (2019). Intravitreal Fluocinolone Acetonide May Decelerate Diabetic Retinal Neurodegeneration. Investig. Opthalmology Vis. Sci..

[83] Zhang X., Wang N., Schachat A.P., Bao S., Gillies M.C. (2014). Glucocorticoids: Structure, Signaling and Molecular Mechanisms in the Treatment of Diabetic Retinopathy and Diabetic Macular Edema. Curr. Mol. Med..

[84] Babu P.V.A., Sabitha K.E., Shyamaladevi C.S. (2006). Therapeutic effect of green tea extract on advanced glycation and cross-linking of collagen in the aorta of streptozotocin diabetic rats. Clin. Exp. Pharmacol. Physiol..

[85] Meng J.M., Cao S.Y., Wei X.L., Gan R.Y., Wang Y.F., Cai S.X., Xu X.Y., Zhang P.Z., Li H.B. (2019). Effects and Mechanisms of Tea for the Prevention and Management of Diabetes Mellitus and Diabetic Complications: An Updated Review. Antioxidants.

[86] Silva K.C., Rosales M.A.B., Hamassaki D.E., Saito K.C., Faria A.M., Ribeiro P.A., Faria J.B., Faria J.M. (2013). Green tea is neuroprotective in diabetic retinopathy. Investig. Ophthalmol Vis Sci..

[87] Kumar B., Gupta S.K., Nag T.C., Srivastava S., Saxena R., Jha K.A., Srinivasan B.P. (2014). Retinal neuroprotective effects of quercetin in streptozotocin-induced diabetic rats. Exp. Eye Res..

[88] Abu-Amero K.K., Kondkar A.A., Chalam K.V. (2016). Resveratrol and Ophthalmic Diseases. Nutrients.

[89] Li J., Yu S., Ying J., Shi T., Wang P. (2017). Resveratrol Prevents ROS-Induced Apoptosis in High Glucose-Treated Retinal Capillary Endothelial Cells via the Activation of AMPK/Sirt1/PGC-1α Pathway. Oxid. Med. Cell Longev..

[90] Losso J.N., Truax R.E., Richard G. (2010). trans-resveratrol inhibits hyperglycemia-induced inflammation and connexin downregulation in retinal pigment epithelial cells. J. Agric Food Chem..

[91] Woo J.H., Lim J.H., Kim Y.H., Suh S.I., Min D.S., Chang J.S., Lee Y.H., Park J.W., Kwon T.K. (2004). Resveratrol inhibits phorbol myristate acetate-induced matrix metalloproteinase-9 expression by inhibiting JNK and PKC delta signal transduction. Oncogene.

[92] Chen Y., Meng J., Li H., Wei H., Bi F., Liu S., Tang K., Guo H., Liu W. (2019). Resveratrol exhibits an effect on attenuating retina inflammatory condition and damage of diabetic retinopathy via PON1. Exp Eye Res..

[93] Aldebasi Y.H., Aly S.M., Rahmani A.H. (2013). Therapeutic implications of curcumin in the prevention of diabetic retinopathy via modulation of anti-oxidant activity and genetic pathways. Int. J. Physiol. Pathophysiol. Pharmacol..

[94] Gupta S.K., Kumar B., Nag T.C., Agrawal S.S., Agrawal R., Agrawal P., Saxena R., Srivastava S. (2011). Curcumin prevents experimental diabetic retinopathy in rats through its hypoglycemic, antioxidant, and anti-inflammatory mechanisms. J. Ocul. Pharmacol. Ther..

[95] Maugeri A., Mazzone M.G., Giuliano F., Vinciguerra M., Basile G., Barchitta M., Agodi A. (2018). Curcumin Modulates DNA Methyltransferase Functions in a Cellular Model of Diabetic Retinopathy. Oxid Med. Cell Longev..

[96] Chucair A.J., Rotstein N.P., Sangiovanni J.P., During A., Chew E.Y., Politi L.E. (2007). Lutein and zeaxanthin protect photoreceptors from apoptosis induced by oxidative stress: Relation with docosahexaenoic acid. Investig. Ophthalmol Vis Sci..

[97] Gong X., Draper C.S., Allison G.S., Marisiddaiah R., Rubin L.P. (2017). Effects of the Macular Carotenoid Lutein in Human Retinal Pigment Epithelial Cells. Antioxidants.

[98] Li S.Y., Lo A.C.Y. (2010). Lutein protects RGC-5 cells against hypoxia and oxidative stress. Int. J. Mol. Sci..

[99] Landon R., Gueguen V., Petite H., Letourneur D., Pavon-Djavid G., Anagnostou F. (2020). Impact of Astaxanthin on Diabetes Pathogenesis and Chronic Complications. Mar. Drugs..

[100] Kowluru R.A., Menon B., Gierhart D.L. (2008). Beneficial effect of zeaxanthin on retinal metabolic abnormalities in diabetic rats. Investig. Ophthalmol Vis Sci..

[101] Keegan G., Pardhan S., Chichger H. (2020). Lutein and zeaxanthin attenuates VEGF-induced neovascularisation in human retinal microvascular endothelial cells through a Nox4-dependent pathway. Exp Eye Res..

[102] Ying Z., Kampfrath T., Sun Q., Parthasarathy S., Rajagopalan S. (2011). Evidence that α-lipoic acid inhibits NF-κB activation independent of its antioxidant function. Inflamm. Res..

[103] Kowluru R.A., Odenbach S. (2004). Effect of long-term administration of alpha-lipoic acid on retinal capillary cell death and the development of retinopathy in diabetic rats. Diabetes.

[104] Derosa G., D’Angelo A., Romano D., Maffioli P. (2016). A Clinical Trial about a Food Supplement Containing α-Lipoic Acid on Oxidative Stress Markers in Type 2 Diabetic Patients. Int. J. Mol. Sci..

[105] Shang F., Lu M., Dudek E., Reddan J., Taylor A. (2003). Vitamin C and vitamin E restore the resistance of GSH-depleted lens cells to H_2_O_2_. Free Radic. Biol. Med..

[106] Miao X., Sun W., Miao L., Fu Y., Wang Y., Su G., Liu Q. (2013). Zinc and diabetic retinopathy. J. Diabetes Res..

[107] Moustafa S.A. (2004). Zinc might protect oxidative changes in the retina and pancreas at the early stage of diabetic rats. Toxicol Appl. Pharmacol..

[108] Lee S.H., Jouihan H.A., Cooksey R.C., Jones D., Kim H.J., Winge D.R., McClain D.A. (2013). Manganese supplementation protects against diet-induced diabetes in wild type mice by enhancing insulin secretion. Endocrinology.

[109] González de Vega R., García M., Fernández-Sánchez M.L., González-Iglesias H., Sanz-Medel A. (2018). Protective effect of selenium supplementation following oxidative stress mediated by glucose on retinal pigment epithelium. Metallomics.

[110] Arrigo A., Aragona E., Bandello F. (2022). VEGF-targeting drugs for the treatment of retinal neovascularization in diabetic retinopathy. Ann. Med..

[111] Blinder K., Dugel P., Chen S., Jumper J.M., Walt J.G., Hollander D.A., Scott L.C. (2017). Anti-VEGF treatment of diabetic macular edema in clinical practice: Effectiveness and patterns of use (ECHO Study Report 1). Clin. Ophthalmol..

[112] Aiello L.P., Avery R.L., Arrigg P.G., Keyt B.A., Jampel H.D., Shah S.T., Pasquale L.R., Thieme H., Iwamoto M.A., Park J.E. (1994). Vascular Endothelial Growth Factor in Ocular Fluid of Patients with Diabetic Retinopathy and Other Retinal Disorders. N. Engl. J. Med..

[113] Ciombor K.K., Berlin J. (2014). Aflibercept—A Decoy VEGF Receptor. Curr. Oncol. Rep..

[114] Thurston G., Daly C. (2012). The Complex Role of Angiopoietin-2 in the Angiopoietin-Tie Signaling Pathway. Cold Spring Harb Perspect Med..

[115] Sahni J., Patel S.S., Dugel P.U., Khanani A.M., Jhaveri C.D., Wykoff C.C., Hershberger V.S., Pauly-Evers M., Sadikhov S., Szczesny P. (2019). Simultaneous Inhibition of Angiopoietin-2 and Vascular Endothelial Growth Factor-A with Faricimab in Diabetic Macular Edema. Ophthalmology.

[116] Midena E., Micera A., Frizziero L., Pilotto E., Esposito G., Bini S. (2019). Sub-threshold micropulse laser treatment reduces inflammatory biomarkers in aqueous humour of diabetic patients with macular edema. Sci. Rep..

[117] Midena E., Bini S., Frizziero L., Pilotto E., Esposito G., Micera A. (2019). Aqueous humour concentrations of PEDF and Erythropoietin are not influenced by subthreshold micropulse laser treatment of diabetic macular edema. Biosci. Rep..

[118] Midena E., Bini S., Martini F., Enrica C., Pilotto E., Micera A., Esposito G., Vujosevic S. (2020). Changes of aqueous humor müller cells’ biomarkers in human patients affected by diabetic macular edema after subthreshold micropulse laser treatment. Retina.

